# Prevalence of somatic symptoms among Ebola Virus Disease (EVD) survivors in Africa: a systematic review and meta-analysis

**DOI:** 10.1186/s12889-024-19013-8

**Published:** 2024-06-05

**Authors:** Oscar Onayi Mandizadza, Ropafadzo Tsepang Phebeni, Conghua Ji

**Affiliations:** 1 School of Public Health, Zhejiang Chinese Medical University, Hangzhou, China; 2 Tongji Medical College, Huazhong University of Science and Technology, Wuhan, China

**Keywords:** Ebola Virus Disease (EVD), Survivors, Somatic symptoms, Systematic Review, Meta-analysis

## Abstract

**Background:**

Many Ebola virus disease (EVD) survivors have reported somatic and neuropsychological symptoms after discharge from the Ebola Treatment Unit (ETU). Since the 2014–2016 Ebola epidemic in West Africa, various studies have investigated and identified these symptoms. Evidence on somatic symptoms is widely available in the literature, however, there is no concise overview of the prevalence across different time intervals.

**Methods:**

This meta-analysis was conducted following the (PRISMA) guidelines. A database search was conducted to identify original studies that reported the prevalence of symptoms. The primary outcome measure was the prevalence rate of several somatic symptoms. Results were pooled, and prevalence rates were assessed over time, to elucidate any particular trends.

**Results:**

We included 23 studies (5,714 participants). The pooled prevalence was: arthralgia 50% (95% CI: 41%-59%); headache 44% (95% CI: 36%-52%); myalgia 32% (95% CI: 26%-38%); abdominal pain 27% (95% CI: 15%-39%); fatigue 25% (95% CI: 19%-31%); numbness of feet 16% (95% CI: 14%-18%); numbness of hands 12% (95% CI: 10%-14%) and hearing loss 9% (95% CI: 5%-12%). Prevalence across different time intervals revealed significant patterns. All the symptoms persisted for more than 2 years after discharge except for abdominal pain.

**Conclusion:**

The pooled prevalence rates of somatic symptoms are notably high. Arthralgia and headache are the most prevalent of the symptoms, with hearing loss and numbness in hands and feet being the least. We found that arthralgia, myalgia, and abdominal pain decreased over time. However, headache, fatigue, numbness of hands and feet, and hearing loss increased over time.

## Introduction

Ebola Virus Disease, EVD (formerly known as Ebola Hemorrhagic Fever) is a rare but fatal disease (case fatality rate of 25–90%). It is caused by four ebolavirus strains (Zaire, Sudan, Taï Forest, and Bundibugyo) of the filoviridae family [1]. The majority of past widespread Ebola virus outbreaks were primarily caused by Zaire (24 outbreaks) and Sudan strains (8 outbreaks). The Zaire strain generally exhibits higher virulence compared to the Sudan strain [2], therefore accounting for more deaths compared to other strains [3]. Several African countries have been affected by recurrent outbreaks since its discovery in 1976 (in the Democratic Republic of Congo) [4, 5] with more than 30 outbreaks recorded [3, 6, 7]. In order to monitor the post-Ebola health status of survivors, studies have been conducted in previous years. Rowe et al. (1999), recorded one of the earliest findings on symptoms experienced by EVD survivors [8].

Prior to 2014, there were limited studies on the health challenges faced by EVD survivors due to the small number of survivors. However, the 2014 West Africa outbreak, brought forth a large cohort of survivors. This led to a significant increase in research efforts focused on the well-being of EVD survivors [9–11]. Notably, many of these studies have been conducted in Africa, where almost all Ebola outbreaks occurred.

EVD survivors report somatic and neuropsychological [12] symptoms that emerged weeks to years after discharge from the Ebola Treatment Unit (ETU) [13–15]. Felix Lotsch et al. [16] reviewed the neuropsychological long-term sequelae in 2017, which is the only existing review on neuropsychological long-term sequelae among EVD survivors. On the other hand, many studies have reported somatic symptoms among EVD survivors. Frequently reported somatic symptoms include headache, fatigue, arthralgia, myalgia, hearing loss, abdominal pain, and numbness of hands and feet. Although these somatic symptoms have been widely reported in literature, there is no concise overview on the prevalence of these symptoms. This study, therefore, aims to determine the prevalence of somatic symptoms among EVD survivors after discharge from an ETU and how these prevalence change over time. This will provide a concise summary of the symptom burden, and its progression over time which will further direct future efforts toward prioritizing care to survivors who by reason of their age are in many instances the breadwinners of the affected societies [17].

## Materials and methods

The study was registered in Prospective Register Of Systematic Reviews (PROSPERO): CRD42023427604.

### Search methods for identification

PubMed, Web of Science and Embase were searched by 2 independent authors for articles that reported symptoms experienced by EVD survivors weeks-to-years after discharge from ETU, from the time of database inception to 30 April 2023. References of the included studies were also manually checked to ensure no relevant studies were missed during the search.

### Electronic searches

The following key terms were used in the search, “Hemorrhagic fever, Ebola”, “survivor”, “sequelae”, etc. Table S1 (Supplement material) shows the full search string used in each database. No language or age restrictions were applied. The search followed the PRISMA guidelines [18]. Only studies that quantitatively included the prevalence of the following characteristics were considered: headache, vision problems, fatigue, uveitis, arthralgia, myalgia, hearing loss, abdominal pain, and numbness of hands and feet.

### Selection of studies

Studies were selected by 2 independent authors according to the following eligibility criteria:

- Original studies: Observational studies (cross-sectional or cohort studies) with primary evidence.
- Studies with EVD survivors, confirmed by either an ETU discharge certificate, positive antibody Ebolavirus antibody test (Ig G), or registration in the National Ebola survivor’s databases.
- Outcome of interest: Studies that recorded the prevalence of headache, fatigue, uveitis, arthralgia, myalgia, hearing loss, abdominal pain, numbness of hands, and numbness of feet, experienced by EVD survivors weeks-to-years after discharge from ETU.

### Exclusion criteria

The study aimed to assess specific post-Ebola symptoms; therefore, studies were excluded if they only recorded symptoms by systems, without specifying the prevalence of the above-mentioned specific symptoms. Case series and case reports were also excluded due to the limited sample size.

### Quality assessment

All included studies were observational (Cross-sectional or cohort). The quality was evaluated using the modified Newcastle Ottawa Scale (NOS) for cohort studies and a modified version for cross-sectional studies [19].

### Measures of outcome

The outcomes of interest were the prevalence of the following symptoms: Headache, fatigue, uveitis, arthralgia, myalgia, vision problems, hearing loss, abdominal pain, numbness of hands, and numbness of feet. “Vision problems” was later excluded from the pooled prevalence rate as it was considered non-specific; since different articles referred to different characteristics within the category “vision problems”, limiting generalizability. In addition, uveitis was further excluded, as it depend on specialist assessment rather than self-reported. The pooled prevalence rate was measured for each symptom in Stata version 17. A random-effect model was used to cater for high heterogeneity of the included studies.

### Data extraction and management

Data from the included studies were entered into a customized Excel spreadsheet by O.O.M and checked for potential errors by R.T.P. The following characteristics were extracted from each of the included studies: first author, year of publication, year of outbreak, Ebola species, study design, country of study, number of participants, the median age of participants, percentage of males, and meantime (months) from ETU discharge. Moreover, data on the somatic symptoms (headache, fatigue, vision problems, uveitis, arthralgia, myalgia, hearing loss, abdominal pain, numbness of hands, and numbness of feet) were extracted.

### Subgroup analysis

Subgroups were evaluated using Stata/MP version 17.0 according to the following categories, to assess the notable trends in prevalence of symptoms across different subgroups. Furthermore, in case of heterogeneity, subgroup analysis was performed to elucidate the potential source of heterogeneity. Subgroups were allocated based on the following characteristics:

- Mean Time from ETU discharge: grouped as 6 months, 12 months, 24 months, and more than 24 months.
- Study design: Cross-sectional (CS) and Longitudinal cohort studies (LS).
- Country of study: Sierra Leone (SL), Uganda (UG), Guinea (GQ), Liberia (LR), the Democratic Republic of Congo (DRC), Sudan and Gabon based on the Center of Disease Control (CDC) list of countries affected by Ebola outbreaks [7]. No studies from Sudan and Gabon satisfied the inclusion criteria.
- Quality of studies: High quality and moderate quality studies.

### Meta analysis

We performed a pooled prevalence rate for all the symptoms using the ‘metaprop’ command in Stata 17 [20]. The ‘metaprop’ command is designed to estimate the pooled prevalence rate, incorporating an in-built double arcsine transformation function. This transformation is essential for stabilization of variance in prevalence studies [20, 21]. Furthermore, a meta regression was performed with the following study characteristics as co-variates to elucidate potential contributing factors to high heterogeneity [22]; Mean time from ETU discharge, study design, country of study, gender (predominantly male: % Male > 50% vs. Predominantly female: % Male < 50%), and the Ebolavirus species.

### Assessment of risk of publication bias and sensitivity analysis

Publication bias was evaluated using the Egger’s regression test and also depicted on a funnel plot [23, 24]. One-by-one exclusion of studies comparing the I^2^ value was performed to identify the source of heterogeneity. I^2^ value was used to quantify the statistical heterogeneity [25]. *P* < 0.10 supported the existence of heterogeneity.

## Results

### Results of the search

**Fig. 1:**
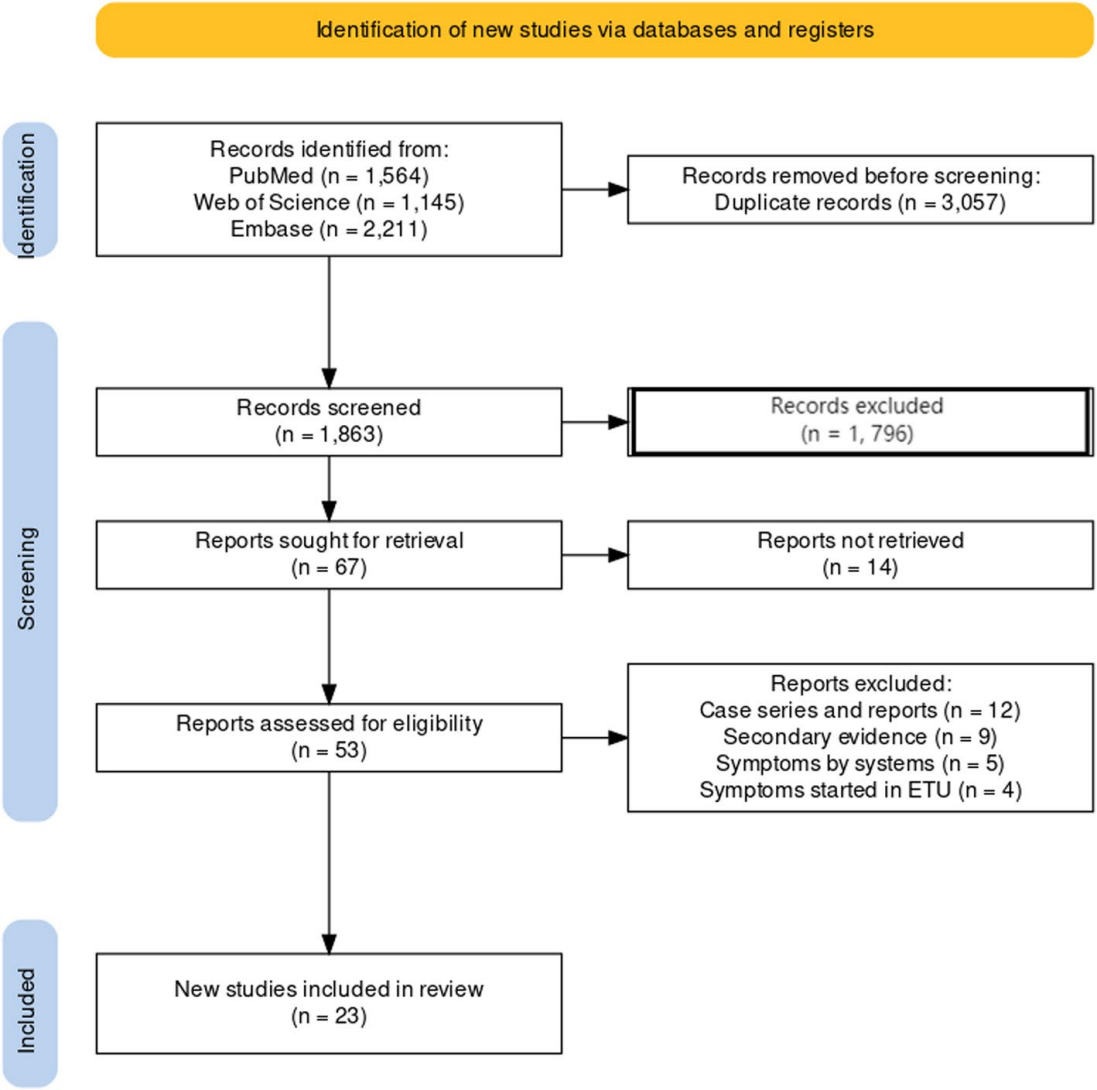
PRISMA Flow chart

A total of 4920 articles were retrieved from the database search. After screening, 23 studies [8, 10, 26–36] with a total population of 5,714 participants were included. A PRISMA flow diagram was created and is presented for reference [37] (Fig. 1).

### Characteristics of included studies

An overview of the included studies is shown in Table 1. The included studies were done in Sierra Leone (11), Liberia (7), and Guinea (3), DRC (1) and Uganda (1). The symptoms experienced by EVD survivors were sparingly distributed across the studies. The prevalence of 10 post-EVD somatic symptoms was recorded weeks to years after discharge from the ETU. Among the 23 included studies, arthralgia (20 studies), headache (16 studies), and myalgia (15 studies) were the three most reported symptoms. Numbness of feet (3 studies) and hands (2 studies) were the least reported. The results of the modified Newcastle Ottawa Score (NOS) evaluation are shown in Table 2 and Table 3. All included studies were moderate to high quality. (shown in Table 2 and Table 3).

**Table 1:** **General Characteristics of included studies**

**Study (First Author)**	**Outbreak**	**Type of Study**	**County of Study**	**Mean Age (Years)**	**% Male**	**Ebola Species**	**Time since ETU discharge (Months)**	**Participants**	**Outcomes **									
									**H**	**F**	**VP**	**U**	**M**	**A**	**N_F**	**N_H**	**H_L**	**A_P**
**A. de St Maurice_2018**	**2014**	**C.S**	**LR**	**33**	**41**	**Z.EBOV**	**6–12**	**329**	**173**	**93**		**16**		**239**	**60**			**148**
**A. I. Qureshi_2015**	**2014**	**C.S**	**GQ**	**38.9**	**67.6**	**Z.EBOV**	**0–6**	**105**	**23**				**28**	**91**			**0**	**33**
**A. K. Rowe_1999**	**1995**	**L.S**	**DRC**	**27**	**31**	**Z.EBOV**	**0–6**	**29**		**2**			**7**	**14**				**4**
**A. K. Rowe_1999**	**1995**	**L.S**	**DRC**	**27**	**31**	**Z.EBOV**	**12–24**	**19**					**9**	**12**				
**A. Tiffany_2016**	**2014**	**L.S**	**SL**	**24.7**	**55.4**	**Z.EBOV**	**0–6**	**166**	**87**	**116**	**94**	**58**		**129**			**5**	**90**
**C. Amuzu_2021**	**2014**	**C.S**	**SL**	**10.09**	**49.1**	**Z.EBOV**	**12–24**	**159**	**138**				**57**	**101**			**33**	
**D. V. Clark_2015**	**2007**	**L.S**	**UG**	**40**	**56**	**B..EBOV**	** > 24**	**49**	**43**	**28**				**17**			**13**	
**D.A Wohl_2022**	**2014**	**L.S**	**LR**	**31.5**	**55.2**	**Z.EBOV**	**12–24**	**326**	**106**	**100**	**59**		**44**	**128**	**52**	**40**	**12**	
**D.A Wohl_2022**	**2014**	**L.S**	**LR**	**31.5**	**55.2**	**Z.EBOV**	** > 24**	**311**	**63**	**38**	**54**		**0**	**49**	**41**	**38**	**9**	
**E. Hereth-Hebert_2017**	**2014**	**L.S**	**GQ**	**26.9**	**44**	**Z.EBOV**	**0–6**	**341**				**46**						
**H. Mohammed_2017**	**2014**	**C.S**	**SL**	**30**	**30.7**	**Z.EBOV**	**0–6**	**115**	**48**		**17**		**46**	**38**			**2**	**28**
**H. Mohammed_2017**	**2014**	**C.S**	**SL**	**30**	**30.7**	**Z.EBOV**	**6–12**	**115**	**60**		**12**		**42**	**45**			**0**	**39**
**H. Mohammed_2017**	**2014**	**C.S**	**SL**	**30**	**30.7**	**Z.EBOV**	**12–24**	**115**	**57**		**3**		**64**	**22**			**0**	**19**
**H. W. Wilson_2018**	**2014**	**C.S**	**LR**	**30**	**35**	**Z.EBOV**	**0–6**	**242**	**10**	**5**	**15**		**12**	**23**				**7**
**H. W. Wilson_2018**	**2014**	**C.S**	**LR**	**30**	**35**	**Z.EBOV**	**6–12**	**242**	**80**	**38**	**55**		**28**	**75**				**19**
**S. C. Ficenec_2022**	**2014**	**L.S**	**SL**	**30.2**	**43**	**Z.EBOV**	** > 24**	**301**									**70**	
**J. D. Kelly_2022**	**2014**	**L.S**	**LR**	**25**	**45**	**Z.EBOV**	**6–12**	**991**	**474**	**180**		**266**	**227**	**467**				
**J. D. Kelly_2022**	**2014**	**L.S**	**LR**	**25**	**45**	**Z.EBOV**	**12–24**	**881**	**283**	**45**		**293**	**110**	**237**				
**J. G. Mattia_2016**	**2014**	**C.S**	**SL**	**29**	**41**	**Z.EBOV**	**0–6**	**277**			**167**	**50**		**210**			**67**	
**J. G. Shantha_2017**	**2014**	**C.S**	**LR**	**38.6**	**86**	**Z.EBOV**	**0–6**	**96**		**20**		**21**		**80**			**1**	
**J. G. Shantha_2022**	**2014**	**C.S**	**SL**	**9**	**60.9**	**Z.EBOV**	**12–24**	**23**				**5**						
**J. T. Scott_2016**	**2014**	**C.S**	**SL**	**35**	**47.7**	**Z.EBOV**	**0–6**	**44**	**21**		**6**		**15**	**12**				**4**
**M. C. Sneller_2019**	**2014**	**L.S**	**LR**	**29**	**45.3**	**Z.EBOV**	**6–12**	**966**	**460**	**178**		**149**	**223**	**459**				
**M. C. Sneller_2019**	**2014**	**L.S**	**LR**	**29**	**45.3**	**Z.EBOV**	**12–24**	**851**	**322**	**75**			**118**	**298**				
**M. C. Sneller_2019**	**2014**	**L.S**	**LR**	**29**	**45.3**	**Z.EBOV**	**12–24**	**860**	**280**	**44**		**172**	**110**	**237**				
**M. Nanyonga_2016**	**2014**	**C.S**	**SL**	**29**	**37**	**Z.EBOV**	**0–6**	**81**	**55**	**24**	**34**		**44**	**66**			**1**	**28**
**N. G. Bond_2021**	**2014**	**C.S**	**SL**	**29.78**	**43.5**	**Z.EBOV**	** > 24**	**375**	**143**				**92**	**146**				
**R. E. G Wadoum_2017**	**2014**	**L.S**	**SL**	**27**	**39.8**	**Z.EBOV**	**6–12**	**246**	**98**		**84**		**182**	**185**				**167**
**R. E. G Wadoum_2023**	**2014**	**C.S**	**SL**	**32.65**	**40.96**	**Z.EBOV**	**0–6**	**83**	**56**		**10**		**62**	**62**			**0**	**8**
**Sam Tozay_2020**	**2014**	**L.S**	**LR**	**31.5**	**55.2**	**Z.EBOV**	**12–24**	**326**	**106**	**100**	**60**		**43**	**128**	**53**	**41**	**12**	
**Yves-Marie Pers_2017**	**2014**	**L.S**	**GQ**	**28.4**	**45**	**Z.EBOV**	**6–12**	**44**		**37**			**27**	**43**				

Outcomes: 1. Headache. 2. Fatigue. 3. Vision Problems. 4. Uveitis 5. Myalgia. 6. Arthralgia. 7. Numbness of feet. 8. Numbness of Hands. 9. Hearing Loss 10. Abdominal pain

*CS* Cross-sectional study, *LS* Longitudinal cohort study, *ETU* Ebola Treatment Unit, *Z.EBOV* Zaire Ebolavirus, *B.EBOV* Bundibugyo Ebolavirus

2014: 2013–2016 Western Africa Ebola Epidemic

2007: Bundibugyo Outbreak

1995: DRC Kikwit outbreak

**Table 2:** Modified Newcastle—Ottawa Quality Assessment Scale (adapted for Cross-sectional Studies)

First Author, year	Selection				Comparability		Outcome			
	**Representative of survivors in the community**	**Justified and satisfactory (including sample size calculation)**	**Satisfactory recruitment rate**	**ETU discharge certificate or Lab confirmed EVD survivors**	**Recorded time from ETU discharge**	**Adjusted for other diseases sex and age**	**Assessed by a physician**	**Objective measures for outcome assessment**	**Appropriate statistical analysis tests (Confidence Interval and p values included)**	**Quality**
M. Nanyonga_2016	yes	yes	yes	yes	NM	NM	yes	NM	NM	Moderate
J. G. Shantha_2017	yes	yes	yes	yes	yes	yes	yes	NM	yes	High
H. Mohammed_2017	yes	yes	yes	yes	yes	yes	yes	NM	yes	High
A. I. Qureshi_2015	yes	yes	yes	yes	yes	yes	yes	NM	yes	High
J. T. Scott_2016	yes	yes	yes	yes	NM	NM	NM	NM	yes	Moderate
J. G. Mattia_2016	yes	yes	yes	yes	yes	yes	yes	yes	yes	High
J. G. Shantha_2022	yes	yes	yes	yes	NM	NM	yes	NM	yes	Moderate
A. de St Maurice_2018	yes	yes	yes	yes	yes	yes	yes	NM	yes	High
C. Amuzu_2021	yes	yes	yes	yes	NM	NM	yes	yes	yes	High
H. W. Wilson_2018	yes	yes	yes	yes	yes	yes	yes	NM	yes	High
N. G. Bond_2021	yes	yes	yes	yes	yes	yes	yes	yes	yes	High
R. E. G Wadoum_2023	yes	yes	yes	yes	NM	NM	yes	yes	yes	High

***NM*** Not Mentioned

**Table 3:** Modified Newcastle—Ottawa Quality Assessment Scale (Cohort Studies)

**First Author, year**	**Selection**				**Comparability**		**Outcome**			**Quality**
	**Representative of survivors in the community**	**Non-exposed cohort from the same community**	**ETU discharge certificate/ Antibody positive**	**Outcome not present at onset of study**	**Presence of a control group**	**Adjusted for other diseases and age**	**Assessed by a physician (or expert)**	**% Lost to follow up unlikely to cause bias** **(< 15% lost to follow-up)**	**Was follow-up long enough for symptom to occur (≥ 3 months)**	
R. E. Guetiya Wadoum_2017	yes	N	yes	N	N	yes	yes	yes	yes	Moderate
Yves-Marie Pers_2017	yes	N	yes	N	N	N	yes	yes	yes	High
E. Hereth-Hebert_2017	yes	NM	yes	NM	N	N	yes	yes	N	Moderate
D. V. Clark_2015	yes	yes	NM	yes	yes	yes	yes	yes	yes	High
S. C. Ficenec_2022	N	yes	yes	yes	yes	yes	yes	yes	yes	High
A. Tiffany_2016	yes	NM	yes	N	N	yes	yes	yes	yes	High
D. A. Wohl_2022	yes	yes	yes	yes	yes	NM	yes	yes	yes	High
M. C. Sneller_2019	yes	yes	yes	yes		yes	yes	yes	yes	High
Sam Tozay_2020	yes	N	yes	yes	N	yes	yes	yes	yes	High
A. K. Rowe_1999	yes	yes	NM	NM	yes	yes	yes	yes	yes	High
J. D. Kelly_2022	yes	yes	yes	yes	yes	N	yes	yes	yes	High

***NM*** Not Mentioned

## The pooled prevalence rate

The pooled prevalence was: arthralgia 50% (95% CI: 41%-59%); headache 44% (95% CI: 36%-52%); myalgia 32% (95% CI: 26%-38%); abdominal pain 27% (95% CI: 15%-39%); fatigue 25% (95% CI: 19%-31%); numbness of feet 16% (95% CI: 14%-18%); numbness of hands 12% (95% CI: 10%-14%) and hearing loss 9% (95% CI: 5%-12%) (Figs. 2, 3, 4, 5, 6, 7, 8, 9 and 10).

**Fig. 2:**
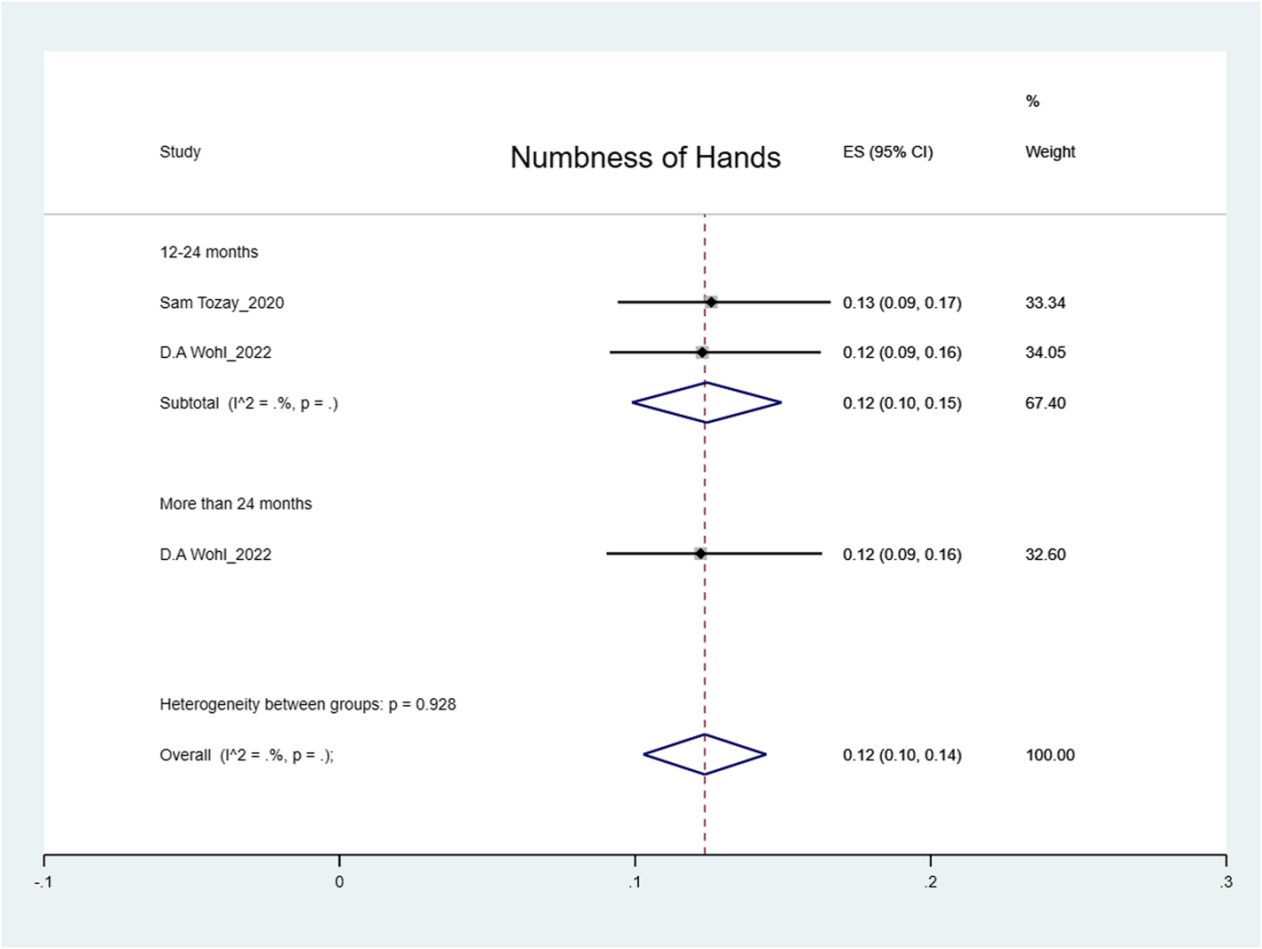
Prevalence of post-EVD, Numbness of hands

**Fig. 3:**
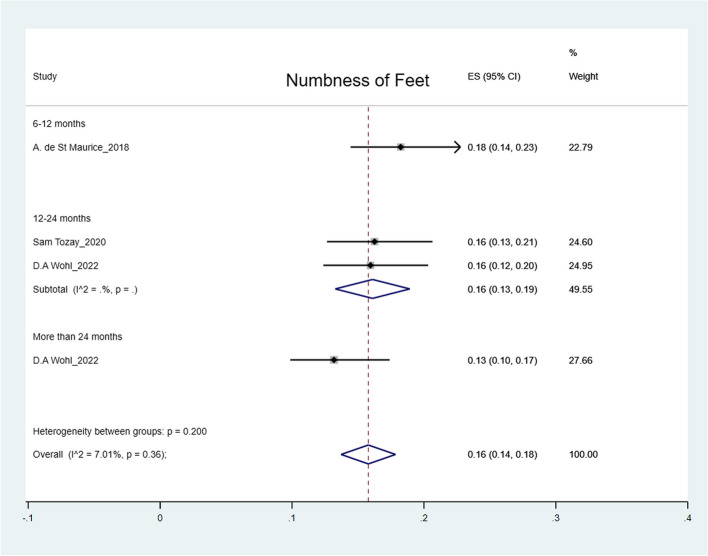
Prevalence of post-EVD, numbness of feet

**Fig. 4:**
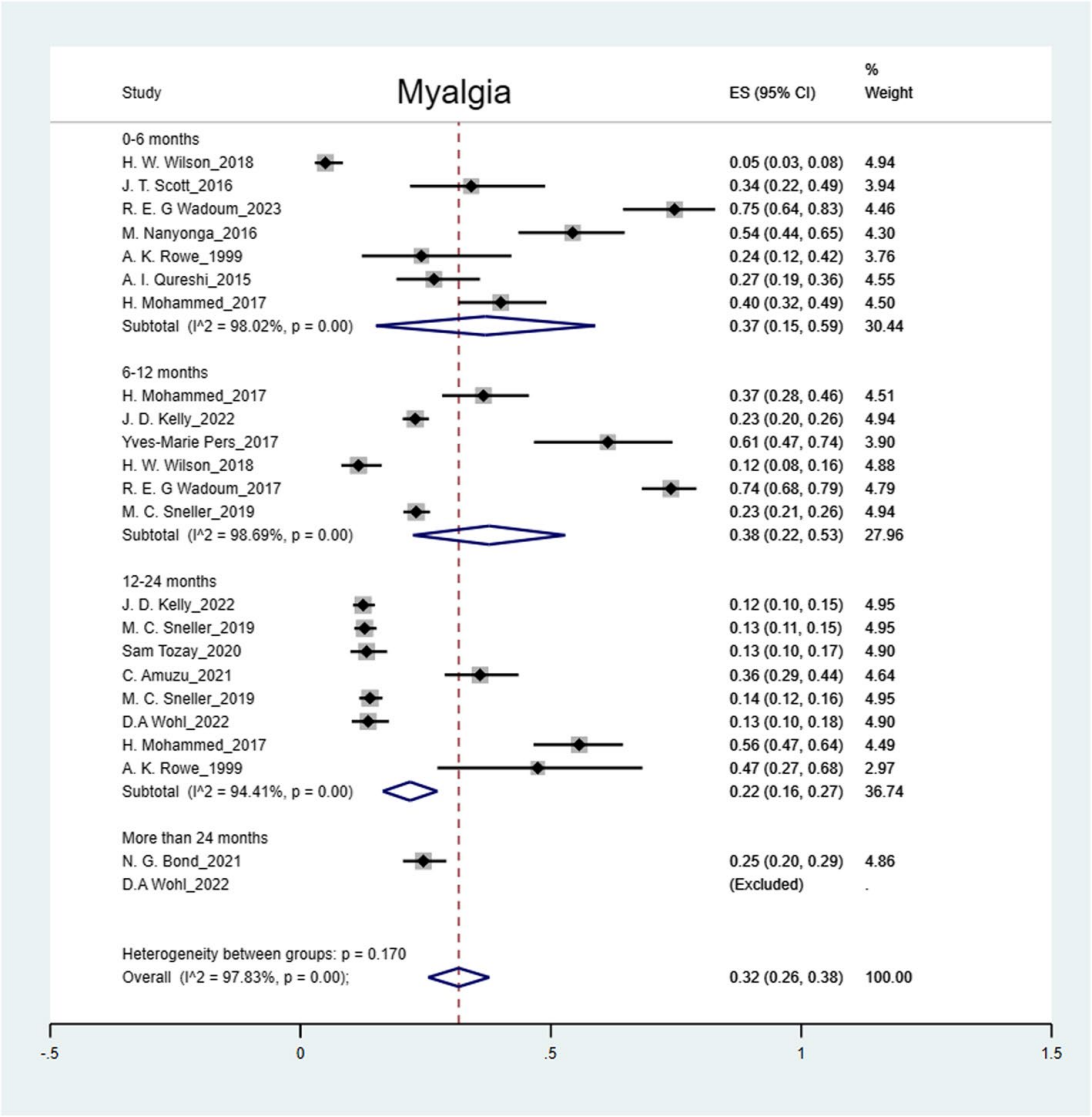
Prevalence of post-EVD, myalgia

**Fig. 5:**
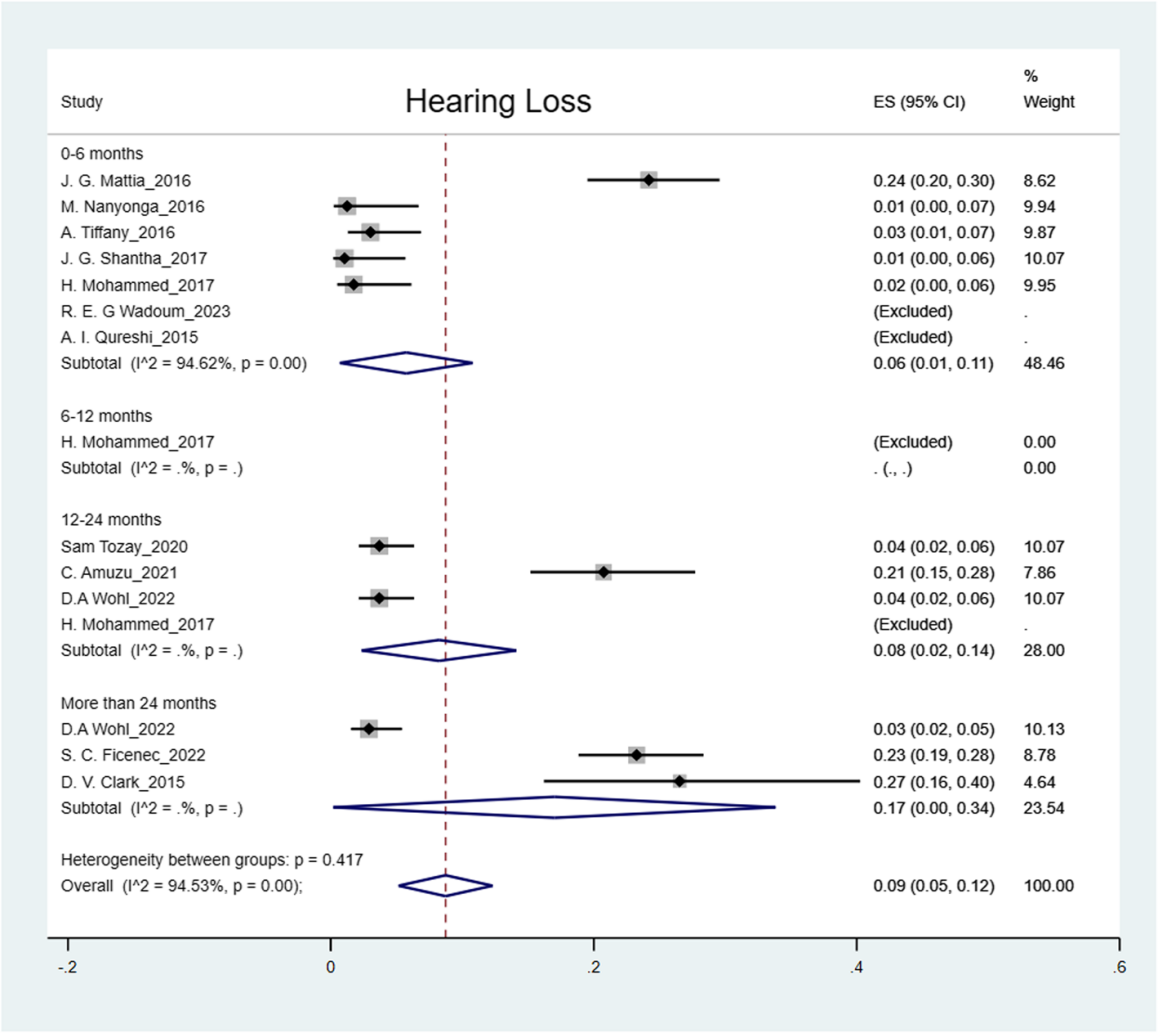
Prevalence of post-EVD, hearing loss

**Fig. 6:**
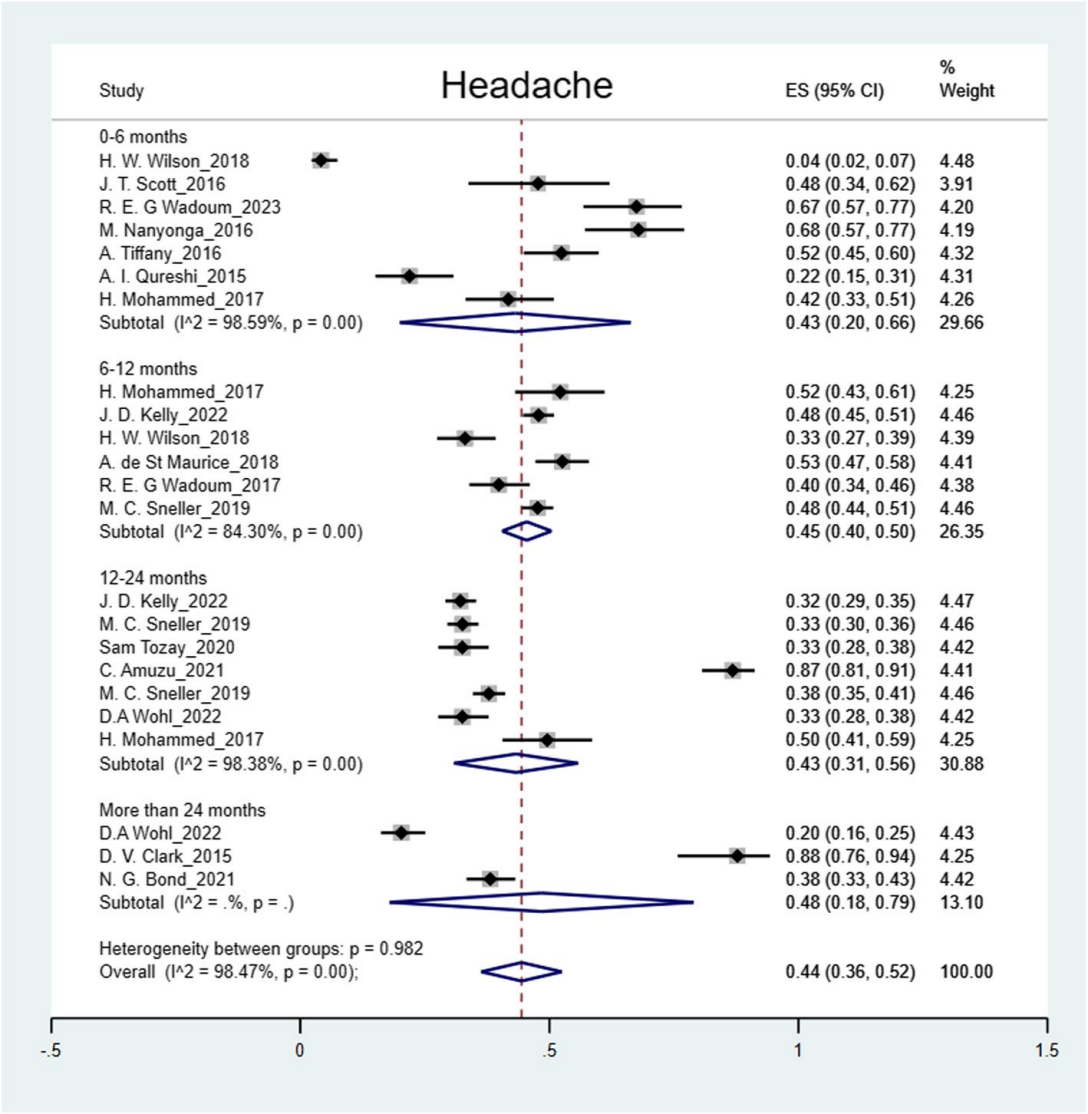
Prevalence of post-EVD, Headache

**Fig. 7:**
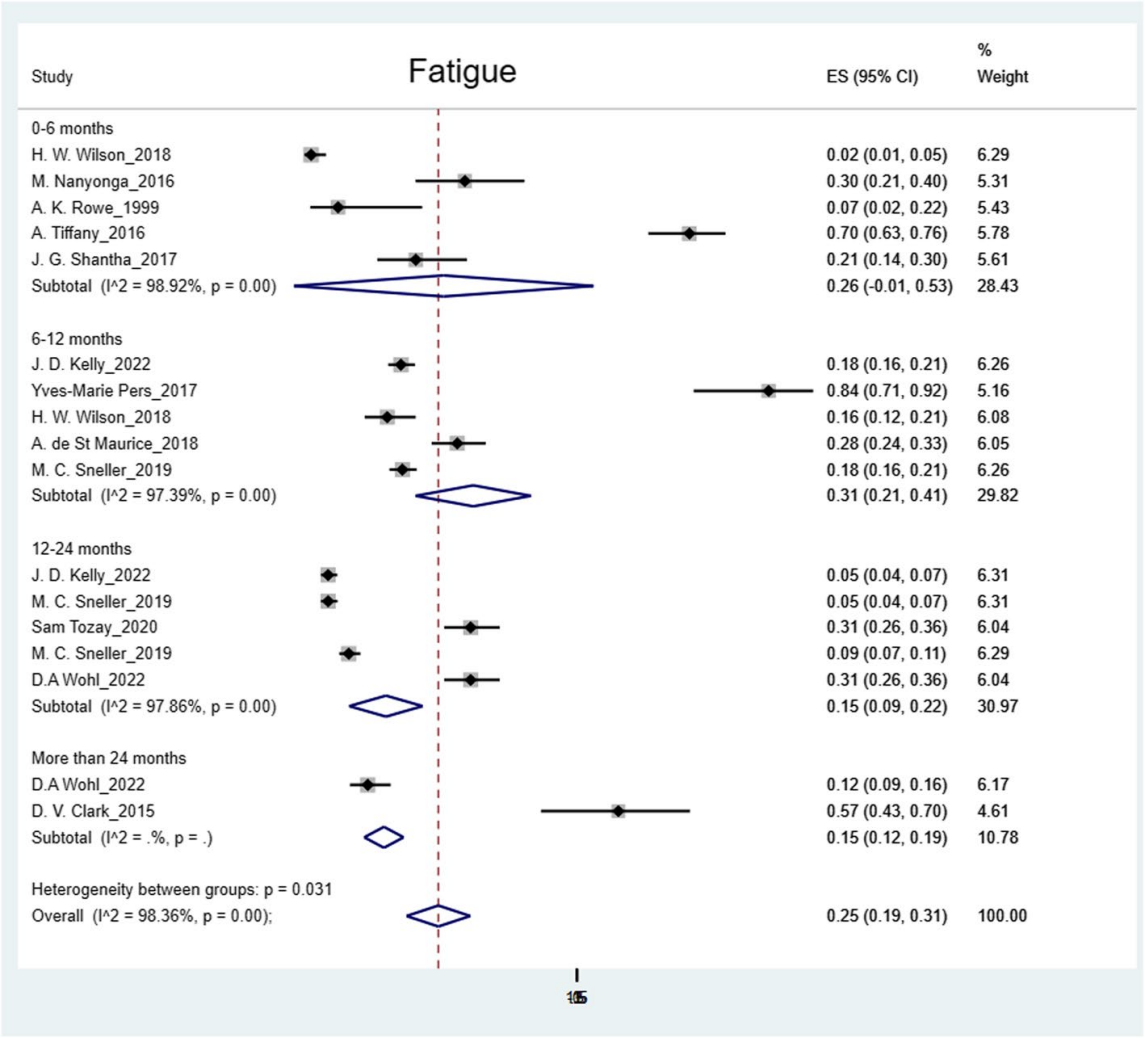
Prevalence of post-EVD, fatigue

**Fig. 8:**
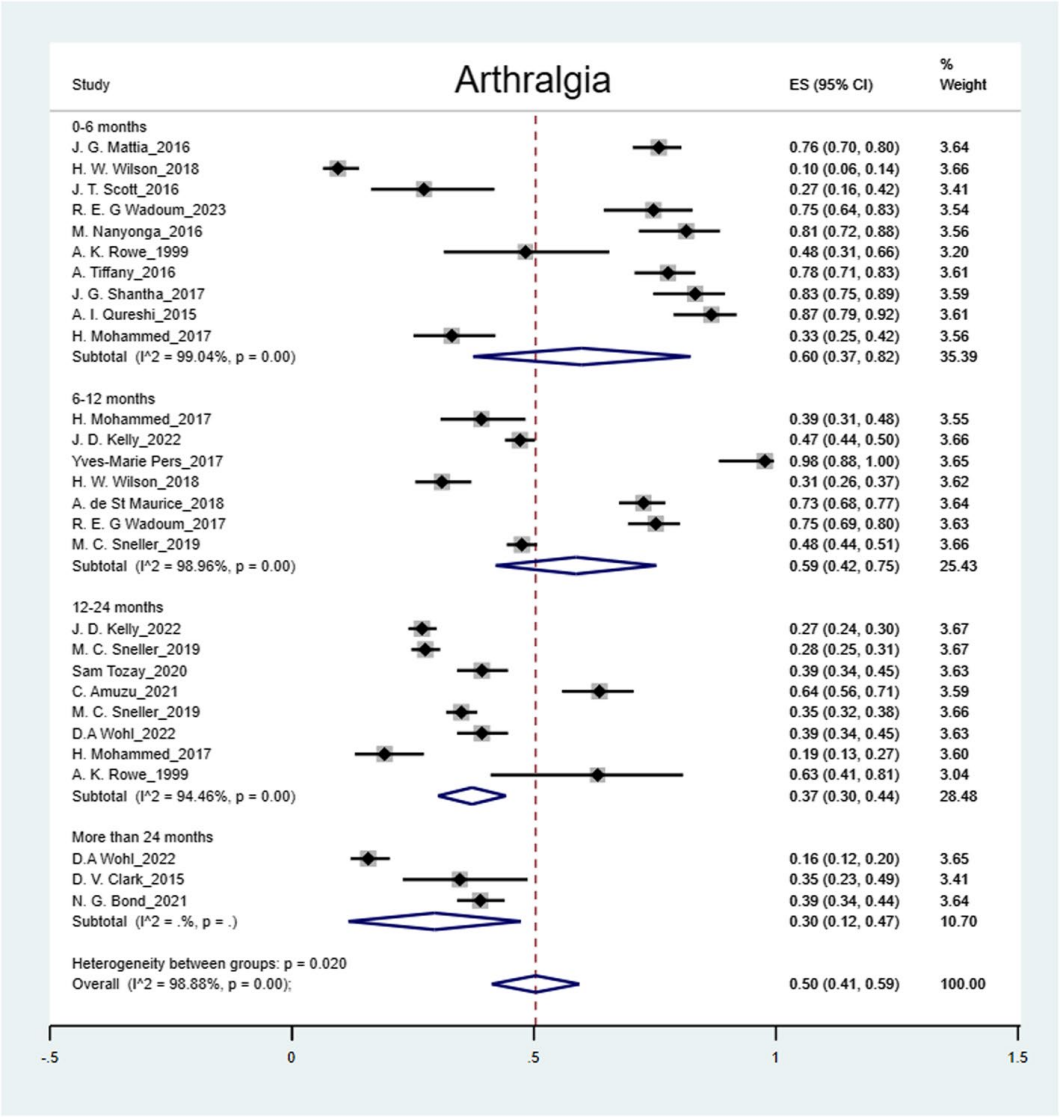
Prevalence of post-EVD, arthralgia

**Fig. 9:**
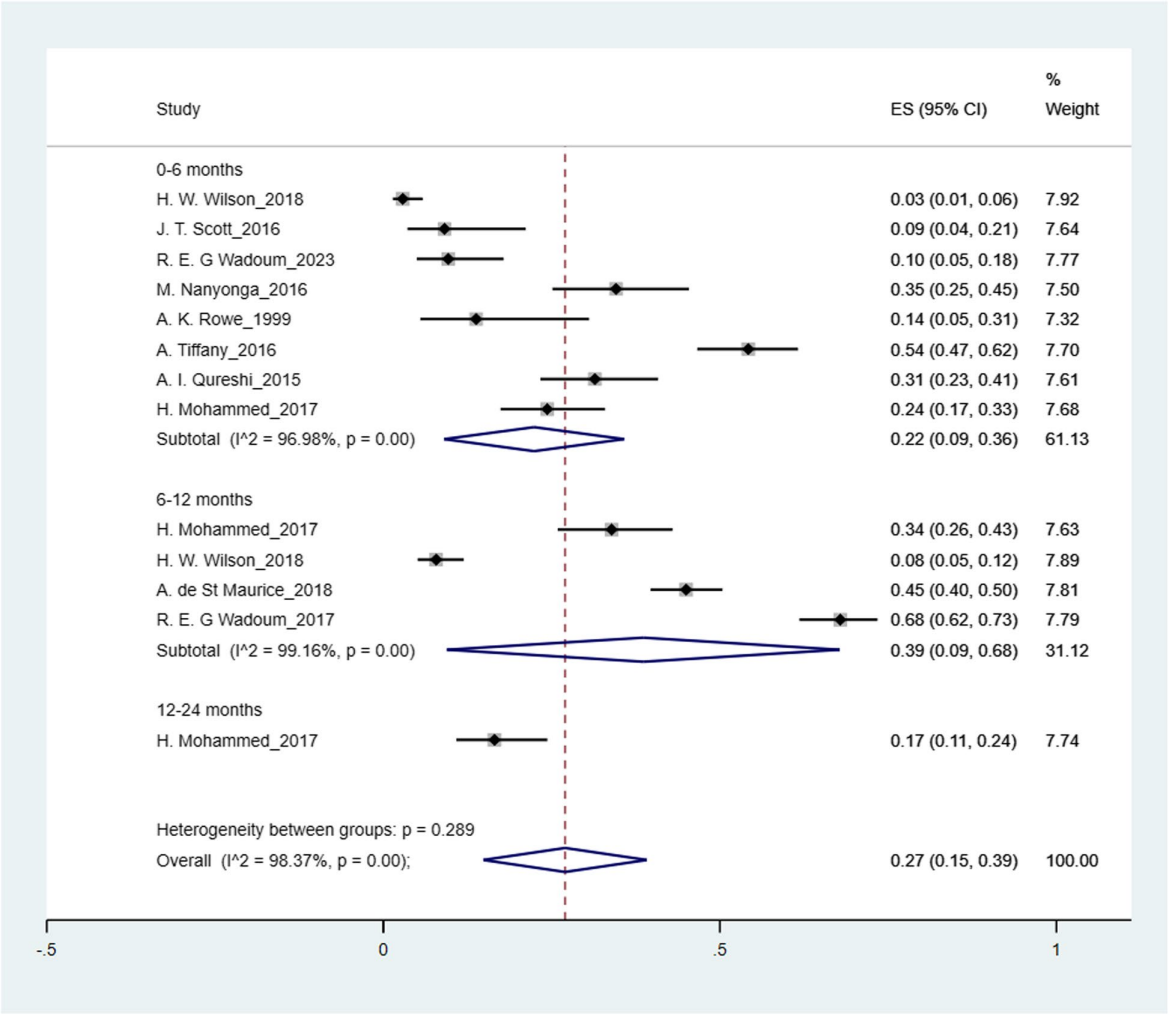
Prevalence of post-EVD, abdominal pain

**Fig. 10:**
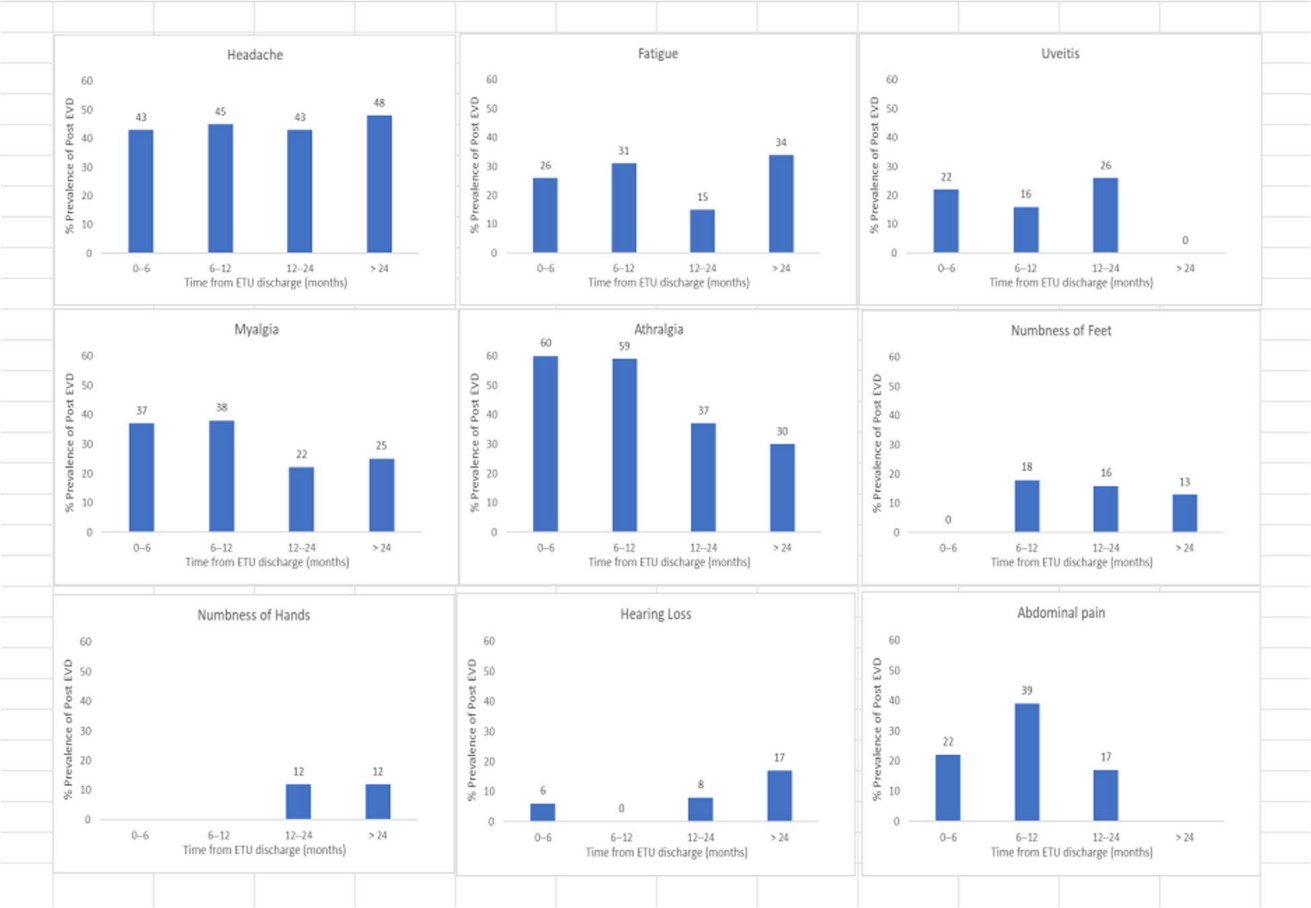
Period prevalence of post-EVD symptoms

## Subgroup analysis

Subgroup analyses were performed for all symptoms to determine any discrepancies across studies, assess the potential source of heterogeneity, and to observe any significant tendencies of these symptoms. Five parameters were assessed: meantime (months) from ETU discharge, study design, quality of study, country of study and presence of control group.

I. Mean time from ETU discharge.
  Results were categorized into four distinct time periods: 0–6 months, 6–12 months, 12–24 months, and more than 24 months post ETU discharge. These intervals excluded the respective lower boundaries (0 months, 6 months, 12 months, and 24 months) to ensure exclusiveness of the time frames (Fig. 10). Within the first 6 months after ETU discharge, symptoms such as arthralgia, myalgia, and fatigue, were more prevalent, while headache, abdominal pain, and hearing loss had lower prevalence compared to the overall prevalence. Data on numbness of hands and feet were missing within the first 6 months (Table 4). Between 6 to 12 months after ETU discharge, higher prevalence rates were reported for arthralgia, headache, abdominal pain, myalgia, and numbness of feet, while fatigue, and hearing loss reported a lower prevalence. No studies reported the prevalence of numbness of hands in the first year after ETU discharge (Table 5). Between 1 to 2 years after ETU discharge, headache, arthralgia, abdominal pain, fatigue, myalgia, numbness of feet, numbness of hands and hearing loss reported a lower prevalence compared to the overall prevalence (Table 6). For symptoms assessed more than 2 years after ETU discharge, headache, fatigue and hearing loss were notably high compared to their respective overall prevalence while arthralgia, myalgia, and numbness of feet and hands had a lower prevalence rate. No studies reported the prevalence of abdominal pain more than 2 years after ETU discharge (Table 7).
II. Study design.
  Crossectional studies reported a notably high prevalence compared to Longitudinal studies in symptoms such as headache (47% vs. 42%), arthralgia (53% vs. 48%), myalgia (36% vs. 27%), and numbness of feet (18% vs. 15%). Conversely, longitudinal studies reported a notably high prevalence compared to Crossectional studies in symptoms such as fatigue (27% vs. 19%), and abdominal pain (46% vs. 21%)
III. Country of Study.
  In the “country of study” subgroup, studies from 5 different countries satisfied the inclusion criteria. There were notable variations in the pooled prevalence across different countries. Studies performed in Uganda reported the prevalence of 3 symptoms (fatigue, headache, and hearing loss), Liberia reported all the symptoms, Sierra Leone reported all the symptoms except numbness of hands and feet, Democratic Republic of Congo reported 4 symptoms (abdominal pain, arthralgia, fatigue, myalgia) and Guinea reported 6 symptoms (abdominal pain, arthralgia, fatigue, headache, and myalgia). A subgroup of studies conducted in Equatorial Guinea [27, 31, 32] reported the highest prevalence in abdominal pain, arthralgia, and fatigue. The study conducted in Uganda by D.V Clark et al. [26] reported the highest prevalence in headache and hearing loss. Among the studies conducted in Sierra Leone, the highest prevalence was reported for abdominal pain and myalgia compared to other countries. Detailed prevalence rates are shown in Table 8.
IV. Quality of study.
  Studies were categorized based on their quality as either high or moderate quality. There were significant variation between the two categories. Notably, moderate quality studies reported a higher prevalence in symptoms such as headache (42% vs. 44%), arthralgia (58% vs. 43%), abdominal pain (34% vs. 16%), myalgia (44% vs. 23%), and fatigue (41% vs. 16%) compared to high quality studies. Conversely, high quality studies recorded a higher prevalence in symptoms such as hearing loss (12% vs. 5%), numbness of hands (13% vs. 12%) and feet (17% vs. 14%) compared to moderate quality studies. Numbness of hands and feet were only reported in high quality studies.

**Table 4:** Prevalence rates based on the mean time since discharge from ETU (subgroup). Prevalence (0-6 months)

Symptom	Prevalence (%)	95% Confidence Interval
Arthralgia	60	37% - 82%
Myalgia	37	15% - 59%
Fatigue	26	1% - 53%
Headache	43	20% - 66%
Abdominal Pain	22	9% - 36%
Hearing Loss	6	1% - 11%

**Table 5:** Prevalence rates based on the mean time since discharge from ETU (subgroup). Prevalence (6-12 months)

Symptom	Prevalence (%)	95% Confidence Interval
Arthralgia	59	42%-75%
Headache	45	40%-50%
Abdominal Pain	39	9%-68%
Myalgia	38	22%-53%
Numbness (feet)	18	14%-22%
Fatigue	31	21%-41%

**Table 6:** Prevalence rates based on the mean time since discharge from ETU (subgroup). Prevalence (12-24 months)

Symptom	Prevalence (%)	95% Confidence Interval
Arthralgia	37	30%-44%
Headache	43	31%-56%
Abdominal Pain	17	10%-23%
Myalgia	22	16%-27%
Fatigue	15	9%-22%
Hearing Loss	8	2%-14%
Numbness (hands)	12	10%-15%
Numbness (feet)	16	13%-19%

**Table 7:** Prevalence rates based on the mean time since discharge from ETU (subgroup). Prevalence (More than 24 months)

Symptom	Prevalence (%)	95% Confidence Interval
Headache	43	31%-56%
Fatigue	15	9%-22%
Hearing Loss	8	2%-14%
Myalgia	25	20%-29%
Arthralgia	30	12%-47%
Numbness (hands)	12	9%-16%
Numbness (feet)	13	9%-17%

**Table 8:** Country of Study vs. Reported symptoms (subgroup)

	**Abdominal pain %**	**Arthralgia %**	**Fatigue %**	**Headache %**	**Hearing loss %**	**Myalgia %**	**Numbness of feet %**	**Numbness of hands %**
**Sierra Leone**	31	55	50	54	12	48	*	*
**Liberia**	18	39	16	34	3	14	16	12
**Equatorial Guinea**	14	92	84	22		44	*	*
**Uganda**	*	*	57	88	27	*	*	*
**DRC**	14	54	7	*	*	34	*	*

^*^Missing data (Symptoms not reported)

***DRC*** Democratic Republic of Congo

## Meta regression

We conducted a meta regression with study characteristics such as mean time from ETU discharge, study design, country of study, gender (predominantly male: % Male > 50% vs. Predominantly female: % Male < 50%), and the Ebola virus species, as covariates. The coefficients for all the covariates were almost zero, and the *p*-values were above the significance threshold of 0.05. The results showed that the covariates were not associated with the prevalence of post-Ebola symptoms (Table 5).

## Results of publication bias assessment and sensitivity analysis

**Fig. 11:**
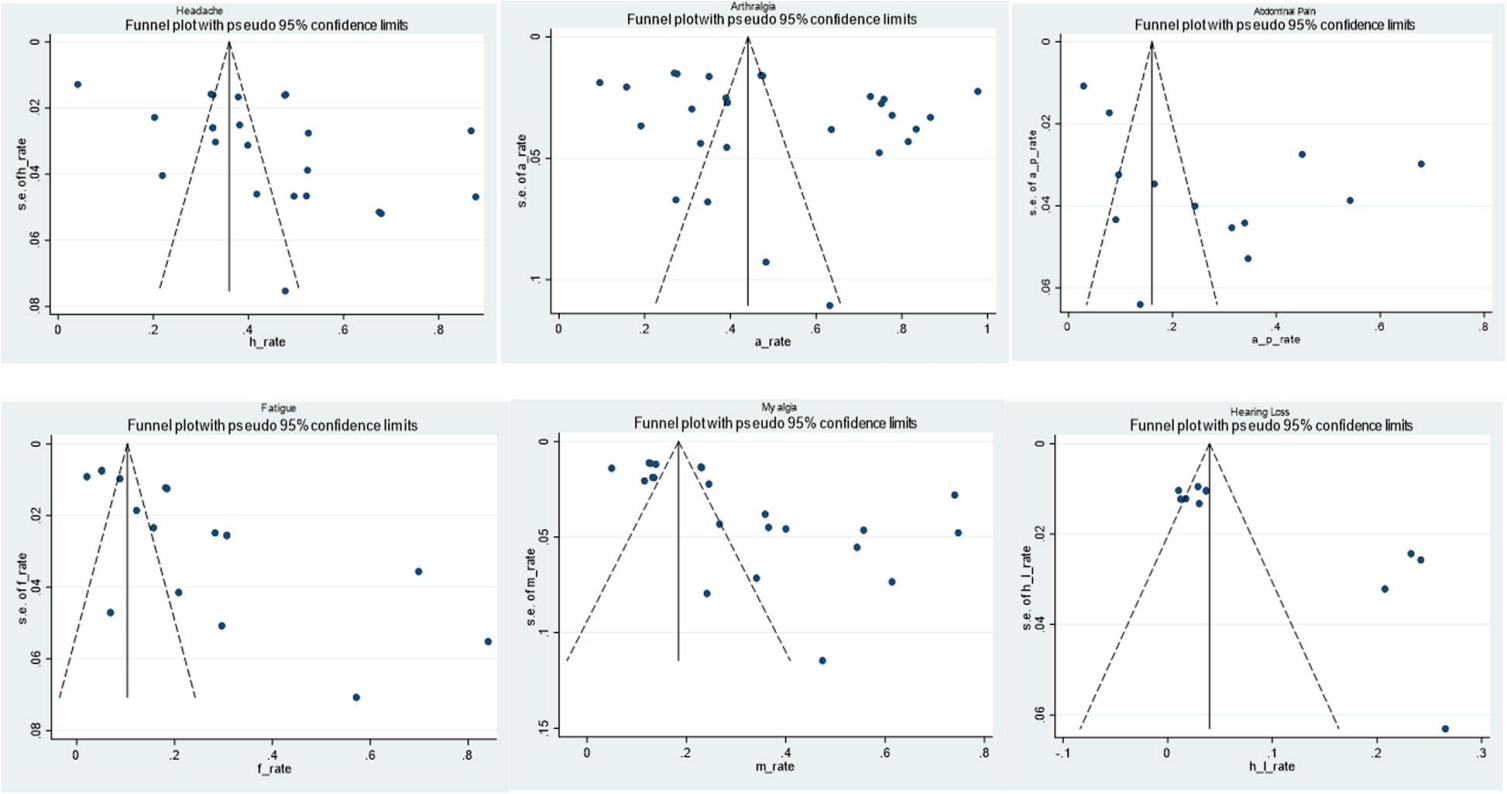
Funnel Plots of post EVD symptoms

We evaluated the impact of publication bias on our findings using the Egger’s regression test and depicted the results using a funnel plot. There was significant publication bias (Egger’s test, *p* = 0.027). The funnel plot is shown in Fig. 11. We performed a sensitive analysis test (omitting studies at a time), to elucidate the effect of individual study on the overall prevalence. Studies by A.K Rowe et al. [5], D.V Clark et al. [26] and R.E.G Wadoum et al. [34], notably influenced the overall pooled prevalence. This impact could be attributed to shorter mean time (2 weeks) from ETU discharge [34], different ebolavirus strain [26] and different employed methodology.

## Discussion

Several studies have reported a notably high prevalence of somatic symptoms among EVD survivors several weeks to year after discharge from an ETU. The impact of somatic symptoms on EVD survivors’ ability to resume to their normal work has been a major concern [38–40]. Therefore, we thought it expedient to estimate the overall prevalence of somatic symptoms experienced by EVD survivors across different time intervals following discharge from the ETU. To our knowledge, this is the only existing systematic review and meta-analysis on post EVD somatic symptoms. 23 studies were included, with a cumulative total of 5,714 EVD survivors (over 30% of all EVD survivors) [41, 42].

Our study showed high prevalence rates of post EVD symptoms (9%-50%). Arthralgia (50%), headache (44%), and myalgia (32%) had the highest prevalence, supporting existing evidence [28, 35, 40, 43, 44]. Generally, most of the somatic symptoms were relatively more prevalent (except for numbness of hands and feet, and hearing loss) within the first year. Numbness of hands and feet were more prevalent more than one year following discharge from ETU. The prevalence of arthralgia and myalgia regressed between 12–24 months. However, headache, fatigue, hearing loss, numbness of hands and feet gradually increased over time. Evidence on prevalence of post EVD symptoms more than 24 months after discharge, remains inconclusive due to limited studies [26, 39, 45], there is need for more research to evaluate the fate of these symptoms. The decline of symptoms over time can be significantly influenced by whether or not EVD survivors received prior treatment, therefore studies performed in clinics were more likely to reflect a significant decrease over time [34, 40]. This observation also highlights the importance of early treatment in mitigating symptom burden among EVD survivors. Moreover, a study conducted by D.V. Clark et al. reported a notably high prevalence of most somatic symptoms. It is worth noting that this study specifically examined post-Ebola Virus Disease (EVD) symptoms among EVD survivors of the Bundibugyo strain, rather than the Zaire ebolavirus, making it the only included study with this particular focus. Therefore, variation in prevalence possibly exist across different Ebola strains [38]. H. Mohammed et al., explored the link between febrile presentation and post EVD symptoms, presuming that febrile presentation could be indicative of another active infection (by other pathogens). Interestingly, the febrile group showed a significantly higher prevalence of headache and fatigue (weakness) compared to the non-febrile group. Therefore, there could be potential inference to comparison of post-infectious symptoms with post-EVD symptoms [46]. It is therefore essential to consider the inclusion of a control group consisting of individuals with no known exposure to Ebola for improved comparability in future research studies. The prevalence remained significantly high across different subgroups (study design, country of study and study quality). The variations in prevalence across different countries could be attributed to many factors such as number of participants enrolled, healthcare systems and accessibility of health services, socioeconomic status of affected population, different ebolavirus strains [26], cultural beliefs among many other. Heterogeneity was potentially due to variations in age, sex, study population, and use of different methodology across studies.

Several mechanisms have been proposed as potential causes of post Ebola symptoms. First, ebolavirus can accumulate in immune-privileged areas such as the testis, central nervous system (CNS), and ocular fluids leading to inflammation (due to reactivation) and direct tissue damage [47]. Viral persistence in ocular fluid and CNS can cause eye and neurological damage [48], leading to neurological [49] and ocular symptoms. Second, ebolavirus causes a robust immunological reaction during acute infection. This leads to an excessive secretion of cytokines, such as tumour necrosis factor (TNF-α), interleukin 6 and interferons which can alter the immunological function [50]. Third, N. G. Bond et al. [45], also described overlapping symptom clusters among EVD survivors, proposing a common underlying mechanism. More evidence is required. Fourth, the healthcare systems, accessibility of health services, socioeconomic status, and prior health status of affected population, could be contributing to the persistence of post Ebola symptoms. EVD survivors in other affluent societies (United States of America) reported complete recovery within a short time [51, 52]. Poor prior health status could impact the duration of the recovery stage.

Scarcity of data on the post-somatic symptoms in EVD survivors has made it difficult to comprehensively understand the underlying mechanisms behind these persistent symptoms. However, the global impact of the COVID-19 pandemic has provided more data for research on post-viral sequelae. Researchers have extensively documented the long term post-COVID-19 symptoms including fatigue and neurological impairments, which are quite similar to the complaints in EVD survivors. The extensive data available on COVID-19 could provide some insights into potential mechanisms and management strategies for both groups of survivors [53].

Our study had several limitations. First, only few studies mentioned the participants’ prior medical history [30, 54, 55]. It is suspected that self-treatment methods might have been used [56], however, none of the studies reported whether or not there was prior treatment by over the counter (OTC) drugs (or other means). Prior treatment can influence the duration of the symptoms. Second, although most studies reported the male-to-female proportions, only one study separated the symptoms experienced by men from those experienced by female participants. Furthermore, variation existed in included age groups among the included studies. Majority encompassing both pediatric and adult participants. These studies reported their results collectively, without differentiating between age groups. Hence, we were unable to assess for heterogeneity across studies based on age, and sex, which are major potential sources of heterogeneity. Future studies should improve on this. Third, some studies were conducted at designated clinics that provided free treatment services which may have led to a potential overestimation of the prevalence rates, as the availability of free services could attract a larger number [30, 33, 57]. Fourth, individual perception of health can vary and might influence whether or not a particular symptom is reported. Therefore, the prevalence rates reported across studies might have been impacted (over or under-estimated) by individual reporting biases. Fifth, Ebola outbreaks occurred in low-resource communities, which previously had limited access to health-care facilities. Therefore, the observed post-Ebola health outcomes could have been influenced by prior health status of the community. Despite the aforementioned limitations, our findings contribute valuable evidence that can serve as a foundation for further research to elucidate the precise mechanisms underlying post-Ebola symptoms. Regions affected by recurrent Ebola outbreaks often rely on agriculture, mining, and trade as their primary means of livelihood [17]. Moreover, it is evident that the majority of these symptoms have a significant impact on individuals' daily activities and overall quality of life. Since most EVD survivors are within the working-age population, they serve in many instances as the breadwinners of the affected societies. Relatively due to stigma [58] and persistent somatic symptoms, some EVD survivors were unable to resume work [59]. Therefore, there is a need to provide timely intervention to this population.

## Conclusion

A large cohort of EVD survivors complain of somatic symptoms weeks to years after discharge from ETU. The pooled prevalence rates of somatic symptoms are notably high. Arthralgia and headache are the most prevalent of the symptoms, with hearing loss and numbness in hands and feet being the least. We found that arthralgia, myalgia, headache, and abdominal pain decreased over time. However, headache, fatigue, numbness of hands and feet, and hearing loss increased over time.Unlike previous studies, we had a large cumulative total of 5,714 participants. In addition, we uniquely documented the trends in prevalence rates in different time intervals. The prevalence rate of post EVD somatic symptoms is notably high, highlighting the need for optimizing care up to two years after discharge from ETU.

## Data Availability

All data and materials have been included in the manuscript.
